# Insecticidal genes of *Yersinia *spp.: taxonomical distribution, contribution to toxicity towards *Manduca sexta *and *Galleria mellonella*, and evolution

**DOI:** 10.1186/1471-2180-8-214

**Published:** 2008-12-08

**Authors:** Thilo M Fuchs, Geraldine Bresolin, Lisa Marcinowski, Joachim Schachtner, Siegfried Scherer

**Affiliations:** 1 Zentralinstitut für Ernährungs- und Lebensmittelforschung (ZIEL), Abteilung Mikrobiologie, Germany; 2 Lehrstuhl für Mikrobielle Ökologie, Technische Universität München, Weihenstephaner Berg 3, 85354 Freising, Germany; 3 Philipps-Universität Marburg, Fachbereich Biologie-Neurobiologie/Ethologie, Karl-von-Frisch-Str. 8, 35032 Marburg, Germany

## Abstract

**Background:**

Toxin complex (Tc) proteins termed TcaABC, TcdAB, and TccABC with insecticidal activity are present in a variety of bacteria including the yersiniae.

**Results:**

The *tc *gene sequences of thirteen *Yersinia *strains were compared, revealing a high degree of gene order conservation, but also remarkable differences with respect to pseudogenes, sequence variability and gene duplications. Outside the *tc *pathogenicity island (*tc*-PAI^*Ye*^) of *Y. enterocolitica *strain W22703, a pseudogene (*tccC2'*/*3'*) encoding proteins with homology to TccC and similarity to tyrosine phosphatases at its C-terminus was identified. PCR analysis revealed the presence of the *tc*-PAI^*Ye *^and of *tccC2'*/*3'*-homologues in all biotype 2–5 strains tested, and their absence in most representatives of biotypes 1A and 1B. Phylogenetic analysis of 39 TccC sequences indicates the presence of the *tc*-PAI^*Ye *^in an ancestor of *Yersinia*. Oral uptake experiments with *Manduca sexta *revealed a higher larvae lethality of *Yersinia *strains harbouring the *tc*-PAI^*Ye *^in comparison to strains lacking this island. Following subcutaneous infection of *Galleria mellonella *larvae with five non-human pathogenic *Yersinia *spp. and four *Y. enterocolitica *strains, we observed a remarkable variability of their insecticidal activity ranging from 20% (*Y. kristensenii*) to 90% (*Y. enterocolitica *strain 2594) dead larvae after five days. Strain W22703 and its *tcaA *deletion mutant did not exhibit a significantly different toxicity towards *G. mellonella*. These data confirm a role of TcaA upon oral uptake only, and suggest the presence of further insecticidal determinants in *Yersinia *strains formerly unknown to kill insects.

**Conclusion:**

This study investigated the *tc *gene distribution among yersiniae and the phylogenetic relationship between TccC proteins, thus contributing novel aspects to the current discussion about the evolution of insecticidal toxins in the genus *Yersinia*. The toxic potential of several *Yersinia *spp. towards *M. sexta *and *G. mellonella *demonstrated here for the first time points to insects as a natural reservoir for yersiniae.

## Background

The toxin complex (Tc) proteins whose insecticidal potential resembles that of the *Bacillus thuringiensis *Bt-toxin were first purified from *Photorhabdus luminescens *which lives in symbiosis with nematodes [1]. They have also been identified in other insect-parasitizing bacteria such as *Serratia entomophila*, *Xenorhabdus nematophilus*, or *Pseudomonas entomophila *[2,3]. Homologous insecticidal toxin genes are present in most genomes of *Yersinia *strains sequenced so far, including *Y. mollaretii*, seven *Yersinia pestis *strains and three *Y. pseudotuberculosis *strains. They have also been found in *Y. frederiksenii *and in two *Y. enterocolitica *strains, T83 and W22703, for which a genome sequence is not yet available [4-6]. However, *tc *genes are absent in *Y. bercovieri *and in *Y. enterocolitica *strain 8081 [7]. Interestingly, Tc proteins of three *Serratia *species and of *Y. frederiksenii *are plasmid-encoded, indicating that these *sepABC*-like genes are part of a horizontally mobile region [4].

Little is known about the biological role of the *tc *genes in *Yersinia *spp. The genes of the *tc *operons have been classified into three types according to their homology, namely *tcdA*/*tcaAB*/*tccAB *(type [A]), *tcdB*/*tcaC *(type [B]), and *tccC *(type [C]) [8]. Tc proteins have recently been shown to be secreted in a type III-dependent manner in *Y. pestis *[9]. Type [A] and [B] Tc proteins are presumably toxins directed against invertebrate and mammalian gut cells, and the variability in terms of Tc composition and Tc sequences may be due to insect- and tissue-specific activity [8,10]. A role of the Tc proteins from *Y. enterooclitica *strain T83, *Y. pseudotuberculosis *strain IP32953 and *Y. pestis *KIM in mice gut colonization and in the actin cytoskeleton rearrangement of human gut cells and mouse fibroblast cells, respectively, has been reported [5,8,11]. The function of TccC remains unknown, but it has been suggested that TccC homologs could contribute to stable biofilm formation in fleas or combatting yet unknown antibacterial effectors in fleas [12], or that they act as universal activator of, or chaperons for, different toxin proteins [13].

*Y. enterocolitica *was the first member of the *Yersinia *genus for which insecticidal activity has been experimentally demonstrated, and *tcaA *encoding a subunit of the toxin complex was identified to be necessary for this activity [6]. The transcription of *tcaA *in *Y. enterocolitica *is completely repressed at 37°C, but strongly induced at lower temperatures with a maximum at approximately 10°C to 15°C. In contrast to *Y. enterocolitica *W22703, *tcaABC *expression in *Y. pseudotuberculosis *strain IP32953 was observed at 15°C and at 37°C [14]. Upregulation of *tcaA *and *tcaB*, but not *tccC*, upon temperature shift from 37°C to 26°C have been shown in two *Y. pestis *strains [15,16]. The IP32953 Tc proteins are toxic against *M. sexta *larvae when expressed heterologously in *E. coli *[14]. Temperature-independent, but weak oral toxicity of several *Y. pseudotuberculosis *to this tobacco hornworm has been reported. *Y. pseudotuberculosis*, unlike *Y. pestis*, causes acute oral toxicity to fleas [12]. However, when the *tcaAB *gene pair from *Y. pseudotuberculosis *was heterologously expressed in *E. coli*, the lysates did not cause excess mortality in fleas, and a *Y. pseudotuberculosis *mutant deleted of the *tc *genes remained toxic toward the arthropod [8]. This is in line with the finding that two *Y. enterocolitica *strains containing a *tcdB-tccC *gene pair (strain CS080) or lacking any *tc*-like genes (strain 8081) were equally toxic to fleas [12].

The insecticidal potential of a variety of *Yersinia *spp. has not been tested in an insect infection assay, and the correlation of virulence to the presence or absence of *tc *operons in yersiniae is unknown. The phylogenetic relationship of the insecticidal toxins is not well understood. Here, we report a genome-based comparison of the *tc *genes in *Y. enterocolitica *strain W22703, the phylogenetic analysis of *tccC *genes in *Yersinia *species, the *tc*-PAI^*Ye *^distribution among six biotypes, and the insecticidal activity of *Yersinia *spp. towards two model organisms, the greater wax moth *G. mellonella *and the tobacco hornworm *M. sexta*.

## Results

### The *tc*-PAI^*Ye *^in *Yersinia *spp

**Figure 1:**
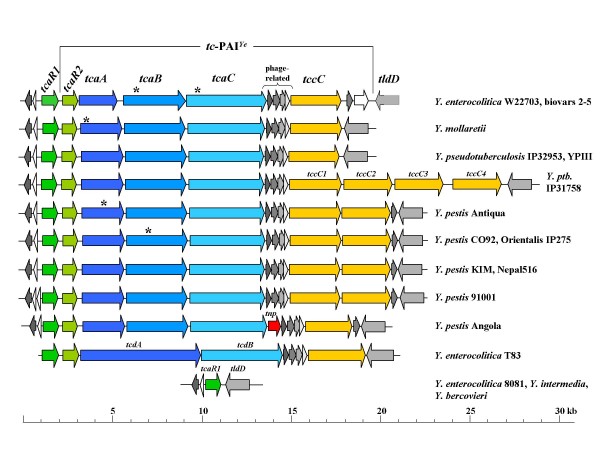
**Comparison of the *tc*-PAI^*Ye *^in Yersiniae**. Three homology groups are depicted, namely *tcaAB*/*tcdA*, *tcaC*/*tcdB*, and *tccC*. *tcdA*/*tcdB *homologous are present only in *Y. enterocolitica *strain T83. *tcaR1 *(left) encoding a regulator and *tldD *encoding a putative DNA gyrase modulator (right, checkered) mark the island insertion site common for all *Yersinia *strains that harbour *tc *homologues. Identically coloured arrows mark homologous genes. A transposase-like gene (*tnp*) is present in the genome of *Y. pestis *Angola (black arrow). The overall gene organisation is similar for all strains harbouring insecticidal determinants, but differences with respect to gene homology, hypothetical ORFs, the presence of transposase-like elements and the number of *tccC *genes are also visible. Gene lengths and intergenic regions are in scale. Asterisks mark frameshifts. With the exception of *tcaC*, all frameshifts result in two ORFs. *Y. ptb*., *Y. pseudotuberculosis*.

Chromosomal loci encoding Tc proteins have first been sequenced from *P. luminescens*, *S. entomophila *and *X. nematophilus*. In the past few years, the genome sequences of several *Yersinia *strains became available. Most of them, namely seven *Y. pestis *strains, three *Y. pseudotuberculosis *strains and *Y. mollaretii*, carry a common *tc *gene cluster termed the *tc *pathogenicity island of yersiniae (*tc*-PAI^*Ye*^). Further DNA fragments encoding insecticidal toxins were detected in the genomes of two *Y. enterocolitica *strains W22703 and T83 [5,6], and on a plasmid of *Y. frederiksenii *strain 49 [4]. A comparison of the chromosomal loci of yersiniae containing *tc *homologues is shown in Fig. 1. The length of the sequences ranges from ~17 kb to ~26 kb. This variation is mainly due to the presence of one to four *tccC *(*1*-*4*) homologues. In all cases, *tc*-PAIs^*Ye *^are inserted into an equivalent location with respect to the common *Yersinia *genome backbone [8], namely between the genes encoding a putative DNA-binding transcriptional regulator and a putative DNA gyrase modulator. These genes are YE3797 (*tcaR1*) and YE3798 (*tldD*) with respect to the chromosomal sequence of *Y. enterocolitica *strain 8081. The overall organisation is similar for all strains, including a second gene, *tcaR2 *that encodes a homolog of LysR-like regulators, followed by genes belonging to the homology types *tcaAB*/*tcdA*, *tcaC*/*tcdB*, and *tccC*. *tccC *is separated from *tcaC*/*tcdB *by two small phage-related genes and two ORFs of unknown function. According to the classification of Waterfield *et al*. [8] described above, all *tc *genes belong to *tcd *operons. Beyond these similarities, several differences were revealed by homology analysis and re-annotation. *Y. enterocolitica *strain W22703 is characterised by a 2034 bp sequence located between *tccC *and YE3798 (*tldD*) harbouring two ORFs of unknown function, one of which is also present in the *Y. pestis *genomes. A transposon-related sequence (*tnp*) was identified in front of the first phage-related gene of *Y. pestis *Angola. Frameshifts (asterisks in Fig. 1) are present in *tcaC *of strain W22703, in *tcaA *of strain Antiqua and of *Y. mollaretii*, and in *tcaB *of strains W22703, Orientalis IP275 and CO92.

### Homologues of *tccC *located outside *tc*-PAI^*Ye*^

By screening a Tn*5 luxCDABE *reporter library of strain W22703 for genes induced upon low-temperature [17], we identified a transposon insertion located outside the *tc*-PAI^*Ye*^. A 4,595 bp sequence encompassing the transposon insertion site was derived, revealing two strain-specific ORFs termed *tccC2' *(1083 bp) and *tccC3' *(1680 bp) due to homologies to other *Yersinia tccC *loci. Obviously, a frameshift had splitted a *tccC *homologue into two ORFs. Exploring the available genome sequences of *Yersinia *strains, two additional *tccC *genes located outside the *tc*-PAI^*Ye *^were identified in *Y. pestis *strains Antiqua, CO92, Nepal516, Orientalis IP275 and 91001, and in *Y. pseudotuberculosis *IP32953, and one further *tccC *gene in *Y. pestis *strains KIM and Angola, and in *Y. pseudotuberculosis *IP31758. A truncated *tccC2*' gene with a 1953 bp deletion in comparison to *tccC2 *of strain IP32953 is present in the genome of *Y. pseudotuberculosis *YPIII. *tccC2' *and *tccC3' *of strain W22703 are located between two genes encoding a lipid A biosynthesis lauroyl acyltransferase, HtrB (YE1612), and a putative membrane protein (YE1611) of strain 8081. In contrast, the non-clustered *tccC *loci of all other strains are inserted into two equivalent locations on the common *Yersinia *backbone. These locations are exemplified as between the *Y. pestis *CO92 genes YPO2379/YPO2381 encoding an N-ethylmaleimide reductase and a lactoylglutathione lyase, and between YPO2311/YPO2313 coding for the insertion element IS1541 and a hypothetical protein. In addition, domain structure of TccC with similarity to a protein tyrosine phosphatase of undefined specificity was identified in the sequence of *Y. pestis *CO92 TccC2 and, albeit with lower probability, of W22703.

**Figure 2:**
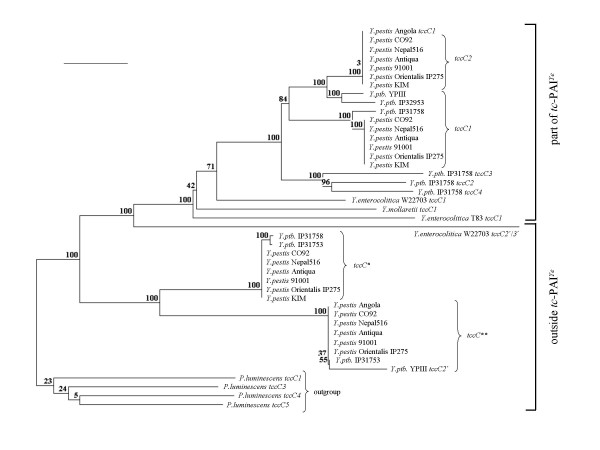
**Cladogram based on 39 TccC amino acid sequence data**. Four TccC sequences of *P. luminescens *subspecies *laumondii *strain TT01 served as outgroup. The phylogenetic analysis was performed with the neighbour-joining method and calculated using the two parameter model of Kimura [31]. Values on each branch indicate the occurrence (%) of the branching order in 500 bootstrapped trees. *insertion site between genes coding for the insertion element IS1541 and a hypothetical protein, **insertion site between genes encoding a N-ethylmaleimide reductase and a lactoylglutathione lyase. The frameshift of *tccC *in strain W22703 was not considered here; hence, one coherent amino acid sequence was used for the alignment. Bar represents 0.1% sequence divergence. *Y. ptb*., *Y. pseudotuberculosis*.

A total of 39 TccC amino acid sequences, derived from yersiniae genes either located within or outside the *tc*-PAI^*Ye*^, was compared here by a ClustlX alignment. The cladogram of the yersiniae TccC proteins *tc*-PAI^*Ye *^exhibits a significant sequence variability between island- and non island-encoded TccCs (Fig. 2). The TccC proteins expressed from loci outside the *tc*-PAI^*Ye *^compose two groups that are characterised by the two common insertion sites as described above. The only exception is TccC2'/3' of strain W22703 that is more closely related to the *tc*-PAI^*Ye*^-encoded TccC proteins. TccC sequences derived from genes located within the *tc*-PAI^*Ye *^show a more complex relationship. One group of TccCs represents TccC1 proteins, a second group TccC2 proteins, indicating a highly conserved linear order of *tccC1 *and *tccC2 *genes in the yersiniae genomes. Interestingly, TccC1 of *Y. pestis *Angola encoded by a *tccC *gene located nearby a transposase-like gene (Fig. 1) appears to be more closely related to TccC2 proteins. Two further sublines that show a higher sequence variability are represented by TccC2-4 of *Y. pseudotuberculosis *IP31758, and by TccC1 of *Y. mollaretii *and *Y. enterocolitica *strains W22703 and T83.

### Presence of *tc *genes in *Y. enterocolitica *strains

**Figure 3:**
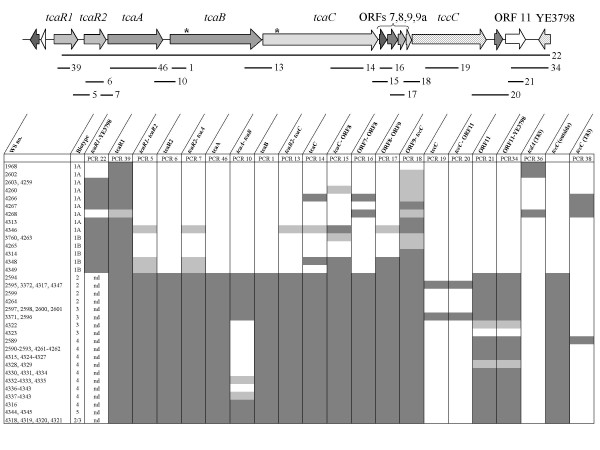
**Distribution of the *tc*-PAI^*Ye *^in *Y. enterocolitica *strains**. Strains investigated are described in Table 3. The lines below the *tc*-PAI^*Ye *^are in scale and mark the fragments amplified by PCR. The PCR numbers correspond to those indicated in Additional file 1. Asterisks mark frameshifts in *tcaB *and *tcaC*. Dark grey: fragment amplification, light grey: biased results, white: no amplification; nd, not defined, e.g. no PCR was performed. See text for further details.

In a previous study, we had studied a restricted number of *Y. enterocolitica *strains by PCR and Southern Blot for the presence of *tcaA*, *tcaB*, and *tcaC *in representatives of five biotypes [6]. Here, we performed a more thorough investigation for the presence, absence, variability and genetic organisation of *tc *genes in a total of 68 *Y. enterocolitica *strains belonging to six biotypes. Chromosomal DNA of all strains was successfully subjected to PCR with oligonucleotides specific for 16S rDNA as control. A series of 22 PCRs designed to amplify intragenic and gene-overlapping fragments was performed (Fig. 3). DNA of strains W22703 and 8081 served as a positive and a negative control. First, *tcaR1 *was confirmed as part of the common yersiniae genomic backbone. The second regulatory gene, *tcaR2*, is present in all strains of biotypes 2–5, but absent in biotypes 1A and 1B. Two different primer combinations failed to amplify *tcaA*-specific fragments from DNA of several strains. Sequencing of four fragments revealed mismatches, and conserved *tcaA *regions were therefore used to design more appropriate oligonucleotides that showed the presence of *tcaA *in biotypes 2–5, but not 1A and 1B (PCR 46). Similar patterns were obtained following amplification of intragenic *tcaB *and *tcaC *fragments (PCRs 1 and 14), with the exception that *tcaC *revealed also to be present in one biotype 1A and one biotype 1B strain. Using oligonucleotides homologous to the 3'-end of *tcaA *and the 5'-end of *tcaB*, the operon organisation was confirmed for most, but not all, biotype 2–5 strains (PCR 10). Negative results in case of PCR 10 predominantly with DNA of biotype 3 strains might be due to a frameshift in *tcaB *similar to that in strain W22703, resulting in an unfunctional and possibly degenerated 5'-end of *tcaB*. Remnants of the phage-related gene cassette which are present in all biotype 2–5 strains could also be amplified from DNA of biotype 1A and 1B strains (ORFs 7,8,9,9a and PCRs 15–18). The results of PCRs 18–20 indicate a highly conserved 5'-region and an otherwise variable *tccC *sequence. We therefore performed a PCR with primers specific for *tccC *of strain T83 and found the respective fragment also in three biotype 1 strains and in one biotype 4 strain. To investigate whether *tc *genes homologous to that of biotype 1A strain T83 are present in other *Y. enterocolitica *strains, PCRs 36 and 37 (data not shown) with oligonucleotides specific for *tcdA *of strain T83 were performed. Fragments of the expected length were obtained using DNA of four biotype 1A strains, a finding that correlates well with the negative result of PCR 22 for fragments overlapping the insertion site of the *tc*-PAI^*Ye*^. Finally, ORF11 is present in all but one biotype 2–5 strains, but absent in most other *Yersinia *spp. (Fig. 1). Taken together, the *tc *operons of strain W22703 are present in all biotype 2–5 representatives tested here. A *tc *gene cluster homologous to that of strain T83 is probably present in biotype 1A strains 1968, 2602 and 4268. In comparison to all other *tc *genes, *tccC *exhibits a higher sequence variability (see chapter above).

### Oral infection of *M. sexta*

Some *Yersinia *spp. besides the three human pathogenic species have not yet been investigated for their insecticidal activity. Oral infection of first-instar *M. sexta *neonates was performed by soaking small blocks of an artificial diet with 50 μl aliquots of overnight cultures grown at 15°C (*Yersinia *spp.) and 37°C (DH5α). *Yersinia *strains applied were *Y. mollaretii*, *Y. aldovae*, *Y. ruckeri*, and four *Y. enterocolitica *strains (W22703, W22703-*tcaA*::Tn*5lux*, 2594, 4466). Their toxicity towards *M. sexta *as percentage of dead larvae five days after infection is shown in Table 1. The experimental setting hampered the reproducibility of the assay, resulting in larger standard deviations in comparison to the *G. mellonella *infection model (see below). The strains *Y. enterocolitica *2594 and *Y. mollaretii *possessing the *tc*-PAI^*Ye *^showed the highest killing rates. Strains without *tc *genes such as *Y. ruckeri *and *Y. aldovae *were less toxic towards *M. sexta*. The only exception was the *tc*-negative *Y. enterocolitica *strain 4466 with a lethality rate of 42%, suggesting the presence of further gut-active insecticidal determinants in its genome. The toxicity of strain W22703 (30% dead larvae) in comparison to that of the *tcaA *knockout mutant W22703-*tcaA*::Tn*5lux *(10%) confirms our recent experiments using concentrated protein extracts instead of viable cells. Taken together, the average killing rates of Table 1 show that the *tc *genes play an important role in the insecticidal activity of *Yersinia *spp. upon oral uptake.

**Table 1:** Oral infection of *M. sexta*

**strain**	***tc*-PAI^*Ye*^**	**total number**	**dead**	**alive**	**dead [%] ± sd**
*Y. enterocolitica *2594	present^2)^	18	15	3	**83 ± 3**
*Y. mollaretii*	present^1)^	25	12	13	**48 ± 9**
*Y. enterocolitica *4466	absent^2)^	36	15	21	**42 ± 15**
W22703	present^2)^	33	10	23	**30 ± 14**
*Y. ruckeri*	absent^2)^	19	4	15	**21 ± 26**
W22703-*tcaA*::Tn*5 lux*	present, but *tcaA *knockout	21	2	19	**10 ± 11**
*Y. aldovae*	absent^2)^	23	2	21	**9 ± 2**
control					

DH5α	absent^1)^	21	1	20	**5 ± 7**

*Yersinia *strains were orally fed to first-instar *M. sexta *neonates. Each experiment was independently performed at least three times with a minimum of six larvae, and the average dead rates are shown. *Y. enterocolitica *W22703: biotype 2, serotype O:9; *Y. enterocolitica *2594: biotype 2, serotype O:9; *Y. enterocolitica *4466: biotype 1B, serotype O:21. ^1) ^according to the genome sequence, ^2) ^according to PCR analysis performed in this study.

### Subcutaneous infection of *G. mellonella*

For an additional bioassay, we chose the model organism *G. mellonella *that is also used to determine the toxicity of *P. luminescens *and *X. nematophilus*. We infected larvae of this greater wax moth subcutaneously with 1:10 and 1:100 diluted aliquots of *Y. mollaretii*, *Y. kristensenii*, *Y. bercovieri*, *Y. aldovae*, *Y. ruckeri*, and four *Y. enterocolitica *strains (W22703, W22703-*tcaA*::Tn*5lux*, 2594, 4466) incubated at 15°C until they reached stationary phase. After five days, only 10% of the larvae had survived an infection of strains 2594 or 4466. In total, four strains showed high killing rates of 81–90% (Table 2). To determine whether the toxicity towards the insect larvae depends on the presence of Tc proteins, we analysed chromosomal DNA of *Y. ruckeri*, *Y. aldovae*, *Y. kristensenii*, and *Y. enterocolitica *strain 4466 with primers described in Additional file 1 and shown in Fig. 3. No fragments were obtained with primers specific to *tcaR2 *(PCR 6), *tcaA *(PCR 46), *tcaB *(PCR 1), *tcaB-tcaC *(PCR 13), *tcaC *(PCR 14), ORF11-YE3798 (PCR 34), and *tccC *of *Y. enterocolitica *strain T83 (PCR 38). As a control, amplification of a *tcaR1 *fragment which is part of the common *Yersinia *backbone sequence (PCR 39) succeeded with DNA of *Y. aldovae *and *Y. enterocolitica *strain 4466. Therefore, we conclude that the *tc*-PAI^*Ye *^is absent in these strains (Table 2). Thus, only two of the strains exhibiting a killing rate of at least 81%, namely *Y. mollaretii*, and the biotype 2 strain 2594, harbour the *tc*-PAI^*Ye*^, while it is absent in *Y. enterocolitica *strain 4466 and *Y. bercovieri*. On the other hand, *Y. ruckeri *and *Y. aldovae *killed *G. mellonella *larvae in approximately 40–50% of all infection experiments, and the insecticidal activity of *Y. kristensenii *is only slightly higher than that of the control strain *S. enterica *serovar Typhimurium. These three strains do not carry *tc *genes, too. No significant difference (p = 0.21) in the insecticidal activity between W22703 and its *tcaA *knockout mutant could be observed. Plasmid-encoded *sep*-like genes of *Y. frederiksenii *isolate 49 could also not be detected using primers specific for *tcYF1*, *tcYF2 *or *tcYF3 *[see Additional file 1] in bacterial lysates of *Y. enterocolitica *strain 4466, *Y. ruckeri*, *Y. aldovae*, or *Y. kristensenii*. According to BLAST analysis, homologous of type III secretion system (T3SS) genes are present in the genomes of W22703 and the other strains from Table 2 sequenced so far, but the role of T3SS and insect virulence as shown for *Y. pestis *remains to be determined for these strains. Taken together, this data strongly suggests that insecticidal activity of *Yersinia *spp. towards *G. mellonella *upon subcutaneous infection is not caused by Tc proteins, and that yet unknown determinants contribute to the insecticidal activity of *Yersinia *strains towards the insect larvae. The similar lethality of strain W22703 and its *tcaA*-negative mutant not only confirms the assumption of Tc-independent killing, but also of a gut-related TcaA activity following oral infection as demonstrated above and recently suggested [6,8,10].

**Table 2:** Subcutaneous infection of *G. mellonella*

			1:10		1:100		total		
**strain**	***tc*-PAI^*Ye*^**	**total number**	**dead**	**alive**	**dead**	**alive**	**dead**	**alive**	**dead [%] ± sd**
*Y. enterocolitica *2594	present^2)^	79	36	4	34	5	70	9	**90 ± 9**
*Y. enterocolitica *4466	absent^2)^	96	47	1	34	14	81	15	**88 ± 11**
*Y. mollaretii*	present^1)^	64	30	2	24	8	54	10	**84 ± 5**
*Y. bercovieri*	absent^1)^	64	28	8	23	5	51	13	**81 ± 11**
*Y. ruckeri*	absent^2)^	85	33	11	20	21	53	32	**53 ± 21**
*W22703-*tcaA::*Tn*5 lux	*present, but *tcaA *knockout*	*93*	*30*	*25*	*18*	*20*	*48*	*45*	***51 ± 13***
*W22703*	*present*^2)^	*114*	*32*	*25*	*20*	*37*	*52*	*62*	***41 ± 17***
*Y. aldovae*	absent^2)^	68	23	13	4	28	27	41	**41 ± 6**
*Y. kristensenii*	absent^2)^	88	19	27	1	41	20	68	**20 ± 12**

controls									
*S. typhimurium*	absent^1)^	88	10	33	10	35	20	68	**18 ± 9**
DH5α	absent^1)^	63	5	34	2	22	7	56	**13 ± 6**
LB		64					3	61	**5 ± 0**

*G. mellonella *larvae were subcutaneously infected with 5 μl of diluted overnight cultures. Strain W22703 and its *tcaA *knockout mutant confer similar toxicity towards the larvae (depicted in italics). Each experiment was independently performed at least three times, and the average dead rates are shown. *Y. enterocolitica *W22703: biotype 2, serotype O:9; *Y. enterocolitica *2594: biotype 2, serotype O:9; *Y. enterocolitica *4466: biotype 1B, serotype O:21. ^1) ^according to the genome sequence, ^2) ^according to PCR analysis performed in this study.

## Discussion

Two basic methods have been used here to determine the insecticidal potential of *Yersinia *spp., namely the oral application of viable cells and the subcutaneous injection of protein extracts or living bacterial cells. Upon oral application of W22703 and W22703-*tcaA*::Tn*5lux *protein extract to *M. sexta *larvae, we could recently demonstrate the role of TcaA in *Y. enterocolitica *toxicity towards insects [6]. Further five *Yersinia *strains were tested here for the first time with respect to their oral toxicity in the *M. sexta *model (Table 1). The presence of the *tc*-PAI^*Ye *^correlates with a higher toxicity of yersiniae towards larvae of the tobacco hornworm, while strains such as *Y. ruckeri *or *Y. aldovae *lacking the *tc *genes are less insecticidal in this assay. The variable insecticidal activity of strains with *tc *genes might be the result of sequence variations, or the presence of further insecticidal components. Due to a higher toxin concentration, the feeding of protein extracts led to higher lethality rates using strain W22703 [8]. In contrast, subcutaneous infection of *G. mellonella *does not result in a significantly different toxicity of these strains (Table 2). In comparison to *P. luminescens *that causes death of *G. mellonella *larvae within 24 hours following injection of several thousand cells [18], approximately 5 × 10^5 ^*Y. enterocolitica *strain W22730 cells are required to kill *G. mellonella *within five days. The most surprising result of the injection study performed here was the high variability of the insecticidal potential among *Yersinia *strains that is probably caused by *tc*-independent determinants. Examples for factors required for full virulence towards insect larvae are the hemolysin XhlA of *X. nematophila *or the gene *mcf *of *P. luminescens*. [13,19]. In *Y. enterocolitica*, XaxAB, an apoptotic AB toxin, and the putative macrophage toxin MT have been identified as candidates with potential insecticidal activity, but their biological role still remains to be uncovered [20]. The overall results of the *Galleria *bioassay correlate with the finding that among 147 *Yersinia *isolates from the environment, 15.6% were *Y. enterocolitica*, but only 0.7% belonged to *Y. kristensenii *[21].

Although the biological role of Tc proteins has still to be experimentally defined, sequence analysis already revealed several interesting aspects. Regions of significant sequence similarities have been identified in all TcdA-like elements characterized so far [14]. Especially, TcaC is well conserved within the *Yersinia *genus, but TcaB and TcaA show significant sequence variability [8]. When the TccC sequences derived from the *tc*-PAI^*Ye *^of yersiniae were aligned, a high degree of sequence conservation was obtained at amino acids 1–680, followed by a remarkably high sequence diversity [14] as is confirmed by the TccC cladogram (Fig. 2). Some Tc sequences show evidence of undergoing degradation with frameshifts that often result in the splitting of *tc *genes into two separate ORFs (Fig. 1). Frameshifts in *Y. pestis*, especially in *tcaB *of CO92, are discussed as a critical step in the recent evolution of flea-borne transmission in the genus *Yersinia *due to the loss of one or more of those insect gut toxins [12,14]. This data indicates that the *tc *genes of yersiniae may be under diversifying selection [8] which might result in insecticidal proteins with host-specific activity and with varying insecticidal potential.

It has been suggested that the genomes of different strains have taken up different *tc *genes after strain separation [22]. However, the data presented here point to a common *Yersinia *ancestor that has aquired the *tc*-PAI^*Ye*^. The plasmid-encoded Tc proteins in *Y. frederiksenii *and a transposon-like element downstream of *Y. pestis *Angola *tcaC *hint to putative mechanisms that might have played a role during horizontal transfer of insecticidal toxin genes (Fig. 1). This hypothesis is strongly supported by the common insertion site of the *tc*-PAI^*Ye *^that indicates one horizontal gene transfer (HGT) event, by the highly conserved phage-related genes within the *tc*-PAI^*Ye*^, and by a similar gene order including *tccC1 *and *tccC2 *in all islands investigated. Moreover, the cladogram derived from a comprehensive alignment of TccC protein sequences (Fig. 2) essentially reflects the phylogeny of *Yersinia *based on 16S rDNA sequences, including the clonal diversity among *Y. enterocolitica *strains [23]. As an additional insecticidal determinant, *tccC *genes located outside the *tc*-PAI^*Ye *^might have been acquired by a further HGT event following the separation of *Y. pseudotuberculosis *and *Y. enterocolitica*, because all available genomes of the *Y. pseudotuberculosis *and *Y. pestis *subline share two *tccC *insertion sites. Thus, reductive evolution by genetic drift might explain the lack of *tc*-PAI^*Ye *^in several *Yersinia *species and strains (Table 2) as examplified by the identification of rudimentary *tc *genes in biotypes 1A and 1B (Fig. 3).

## Conclusion

The prevalence of the *tc*-PAI^*Ye *^in many genomes, its proven functionality in *Y. enterocolitica *and *Y. pseudotuberculosis*, as well as the common insecticidal potential of *Yersinia *spp. towards *M. sexta *and *G. mellonella*, hints to insects as yet unknown host organisms of yersiniae. This is in line with the hypothesis that environmental predators such as nematodes or insect larvae play a role in the evolution of pathogens [22,24]. The *tc*-PAI^*Ye *^has probably been acquired by an ancestral *Yersinia *strain before the separation of *Y. pestis*, *Y. pseudotuberculosis*, *Y. enterocolitica*, and others. This ancestor strain could then have evolved the ability to exploit invertebrates by the acquisition of further genetic determinants required for the interaction of yersiniae with those hosts [20]. Distinct sequence variation, and reductive evolution especially within the genomes of *Y. pestis *serovars, might have allowed yersiniae to occupy specific ecological niches [22]. The role of the *tc *genes and other insecticidal determinants in proliferation and transmission of the three human pathogenic *Yersinia *species remains to be elucidated in more detail.

## Methods

### Bacterial strains and growth conditions

*Y. enterocolitica *strains used in this study are listed in Table 3. *Y. mollaretii *(CIP 103324), *Y. ruckeri *(CIP 82.80), *Y. bercovieri *(CIP 103323), *Y. aldovae *(CIP 103162) and *Y. kristensenii *(CIP 80.30) were obtained from the Collection Institute Pasteur (Paris, France). Strain W22703-*tcaA*::Tn*5lux *is a tcaA knockout mutant [6].*Salmonella enterica *serovar Typhimurium is the ATCC strain 14028. All cultures were grown in Luria-Bertani (LB) broth (10 g l^-1 ^tryptone, 5 g l^-1 ^yeast extract, and 5 g l^-1 ^NaCl) or on LB agar (LB broth supplemented with 1.5% w/v agar). *E. coli *was grown at 37°C and *Yersinia *strains at 15°C or 30°C. If appropriate, the media were supplemented with the following antibiotics: 50 μg ml^-1 ^kanamycin and 20 μg ml^-1 ^nalidixic acid.

**Table 3:** *Y. enterocolitica *strains used in this study

**WS no**.	**Biotype**	**Serotype**	**Strain**	**Geographic origin**	**Biological origin**
1968	1A	n. d.	MZ0124^a)^	n. d.	Concentrate of whey
4346	1A	O:5	Y755^c)^	France	Pony
2602	1A	O:5	H79/83^b)^	Germany	Man
2603	1A	O:5	H1527/93^b)^	Germany	Man
4259	1A	O:41,43	SZ593/04^b)^	Germany	Baby food
4260	1A	O:41,43	SZ554/04^b)^	Germany	Food
4266	1A	O:4,33	SZ1167/04^b)^	Germany	Man
4267	1A	O:10	SZ671/04^b)^	Germany	Man
4268	1A	O:41,43	SZ634/04^b)^	Germany	Man
4313	1A	O:5	NFO^c)^	New Foundland	Man
4346	1A	O:5	Y755^c)^	France	Pony
3760	1B	O:8	8081^g)^	USA	Man
4263	1B	O:8	SZ506/04^b)^	Germany	Man
4265	1B	O:8	SZ375/04^b)^	Germany	Man
4314	1B	O:8	WA-314^c)^	USA	Man
4348	1B	O:8	Y286^d)^	USA	n. d.
4349	1B	O:13	Y293^d)^	n. d.	n. d.
4466	1B	O:21	209–36/84^b)^	Germany	Man
2594	2	O:9	H692/94^b)^	Germany	n. d.
2595	2	O:9	H621/87^b)^	Germany	Man
2599	2	O:5,27	H280/83^b)^	Germany	n. d.
3372	2	O:9	W22703 ^h)^	n. d.	n. d.
4264	2	O:5,27	SZ1249/0^b)^4	Germany	Man
4317	2	O:9	Y738^c)^	France	Man
4347	2	O:9	Y127^d)^	n. d.	n. d.
2596	3	O:9	H324/78^b)^	n. d.	Pig
2597	3	O:9	H7580/93^b)^	n. d.	n. d.
2598	3	O:9	H7692/93^b)^	n. d.	n. d.
2600	3	O:5,27	H230/89^b)^	Germany	Man
2601	3	O:5,27	H582/87^b)^	n. d.	Man
3371	3	O:1	NCTC 10460^f)^	Denmark	Chinchilla
4322	3	O:3	Y745 ^c)^	Japan	Man
4323	3	O:3	Y746^c)^	Japan	Man
2589	4	O:3	H270/78^b)^	n. d.	Dog feces
2590	4	O:3	H31/80^b)^	n. d.	Pig
2591	4	O:3	H608/87^b)^	n. d.	Man
2592	4	O:3	H450/87^b)^	n. d.	Man
2593	4	O:3	H469/87^b)^	n. d.	Pig
4261	4	O:3	SZ425/04 ^b)^	Germany	Pig tongue
4262	4	O:3	SZ687/04^b)^	Germany	Dog feces
4315	4	O:3	Y-108^c)^	Germany	Man
4324	4	O:3	Y747^c)^	Sweden	Man
4325	4	O:3	Y750^c)^	China	Man
4326	4	O:3	Y751^c)^	Great Britain	Man
4327	4	O:3	Y752^c)^	Brazil	Man
4328	4	O:3	Y753^c)^	New Caledonia	Man
4329	4	O:3	Y754^c)^	New Caledonia	Man
4330	4	O:3	Y755^c)^	South Africa	Man
4331	4	O:3	Y756^c)^	South Africa	Man
4332	4	O:3	Y757^c)^	Hungary	Man
4333	4	O:3	Y758^c)^	Hungary	Man
4334	4	O:3	Y759^c)^	Canada	Man
4335	4	O:3	Y763^c)^	Canada	Man
4336	4	O:3	Y764^c)^	Canada	Man
4337	4	O:3	Y765^c)^	Australia	Man
4338	4	O:3	Y766^c)^	Australia	Man
4339	4	O:3	Y767^c)^	Australia	Man
4340	4	O:3	Y768^c)^	Australia	Man
4341	4	O:3	Y769^c)^	New Zealand	Man
4342	4	O:3	Y770^c)^	New Zealand	Man
4343	4	O:3	Y771^c)^	New Zealand	Man
4316	4	O:3	Y11 ^d)^	n. d.	n. d.
4344	5	O:2a,2b,3	Y772^c)^	France	Hare
4345	5	O:2a,2b,3	Y773 ^c)^	France	Hare
4318	2/3	O:5,27	237 ^c)^	USA	n. d.
4319	2/3	O:5,27	238 ^c)^	Great Britain	n. d.

^a) ^own collection, ^b) ^Institut für Hygiene und Umwelt, Hamburg, Germany; ^c) ^Max von Pettenkofer-Institut, München, Germany; ^d) ^Institut für Mikrobiologie der Bundeswehr, München, Germany; ^e) ^Robert Koch-Institut, Berlin, Germany; ^f) ^NCTC, London, UK; ^g) ^Virginia Miller, St. Louis, USA; ^h) ^Roos Goverde, Utrecht, Netherlands. WS, Weihenstephaner Sammlung. N. d., not defined.

### General molecular techniques

DNA and RNA manipulation was performed according to standard procedures [25]. To isolate chromosomal DNA, 1.5 ml of a bacterial culture was centrifuged, and the sediment was resuspended in 400 μl of lysis buffer (100 mM Tris pH 8.0, 5 mM EDTA, 200 mM NaCl). After incubation for 15 min on ice, 10 μl of 10% SDS and 5 μl of proteinase K (10 mg/ml) were added, and the sample was incubated overnight at 55°C. The chromosomal DNA was then precipitated with 500 μl of isopropanol, washed in ethanol, dried, and dissolved in 500 μl of TE buffer (10 mM Tris-HCl, 1 mM Na_2 _EDTA, pH 7.4) containing 1 μl of RNase (10 mg/ml). Polymerase chain reactions (PCR) were carried out with Taq polymerase (Fermentas, Vilnius, Lithunia) and the following programme: one cycle at 95°C for 2 min; 30 cycles at 95°C for 10 sec, at the appropriate annealing temperature for 30 sec, at 72°C for 45 sec to 180 sec depending on the expected fragment length; one cycle at 72°C for 10 min. All primers used are listed in Additional file 1. 4 μl of chromosomal DNA (100 ng ml^-1^) was used as template for PCR amplification, and the GeneRuler DNA mix (Fermentas) served as DNA ladder.

### Inverse PCR and DNA sequencing

Identification of the transposon insertion site in mutant W22703-*tccC*(405)::Tn*5lux *was performed as described previously [17]. Briefly, 400 ng chromosomal DNA of the transposon mutant was completely digested with *Cla*I, *Hind*III or *Ssp*I (Fermentas), enzymes were heat-inactivated, and fragments were treated with T4 DNA ligase (Invitrogen, Carlsbad, USA) to allow self-ligation resulting in circular molecules. Inverse PCR [26] was then performed using transposon-specific primers [17], and the resulting fragments were sequenced with primers hybridizing to transposon regions near the O-end and the I-end. Sequencing of the strain-specific DNA was performed following inverse PCR using the restriction enzymes *Hae*III (USB, Cleveland, USA), *Hha*I, *Hind*III, *Hpa*I, *Msp*I, *Mun*I, *Rsa*I, *Ssp*I and *Vsp*I (Fermentas), and primers derived from the sequence already obtained. Sequencing was done by 4 base lab (Reutlingen, Germany) and by MWG-Biotech (Ebersberg, Germany).

### Bioinformatics

Mapping of the mini-Tn*5 luxCDABE *insertion was performed using the *Y. enterocolitica *Blast Server from the Sanger Institute . The reference genome sequence was that of *Y. enterocolitica *8081 (accession numbers AM286415 and AM286416). Sequence assembly was done with Vector NTI Advance™ (Invitrogen, Carlsbad, USA). The resulting sequence was annotated using the NCBI ORF-Finder . Homology searches of predicted proteins were performed by BLAST analysis . Genome sequences of *Yersinia *strains were obtained from the NCBI database and compared using the homepage . Protein sequence alignment was done with the ClustalW program [27], and cladogram was constructed with TREECON [28]. Promoter sequences located upstream of the identified genes were deduced with BPROM . The accession number of the W22703 *tccC2' *and *tccC3' *sequence is AM941739.

### Bioassays

*M. sexta *were reared as described recently [29]. For oral bioassays, bacteria were grown at 15°C (*Yersinia *strains) or 37°C (DH5α) until stationary phase. 50 μl of a culture was applied to 4-mm^3 ^disks of an agar-based artificial diet [30]. The liquid was allowed to soak into the agar block which was then dried under a laminar flow. First-instar *M. sexta *neonate larvae were then placed on the disk and incubated at 22°C. The application of bacterial culture aliquots was repeated after three days, and the larvae mortality was recorded after 5 days.

Larvae of the greater wax moth, *G. mellonella*, were obtained from the Zoo-Fachmarkt (München, Germany), and stored for less than one week at room temperature. Bacterial strains were grown to stationary phase at 15°C (*Yersinia *spp.) or 37°C (*S. enterica *serovar Typhimurium and DH5α) and then diluted 1:10 and 1:100. 5–7.5 μl of each dilution corresponding to approximately 5–7.5 × 10^5 ^and 5–7.5 × 10^4 ^viable cells were subcutaneously injected into larvae of 2–3 cm length and of 90–140 mg weight using a sterilized micro syringe (Hamilton 1702 RN, 25 μl). Infected larvae were then incubated for five days at 15°C, and the numbers of killed and living larvae were enumerated.

## Authors' contributions

TMF compared the *Yersinia *genomes sequences, performed the bioassays, supervised the study, and drafted the manuscript. GB identified the novel *tccC *gene and derived the distribution pattern. LM performed the PCR analysis. JS supported the bioassays. SS contributed to the conception and revised the manuscript. All authors read and approved the final manuscript.

## Supplementary Material

Additional file 1 **Oligonucleotides used in this study.** Click here for file
