# Supplementary material for: Insecticidal genes of Yersinia spp.: taxonomical distribution, contribution to toxicity towards Manduca sexta and Galleria mellonella, and evolution
Source: BMC Microbiol. 2008 Dec 8;8:214. doi: 10.1186/1471-2180-8-214 (PMC2613401; doi:10.1186/1471-2180-8-214)
Supplement: Additional file 1 — Oligonucleotides used in this study. [file 1471-2180-8-214-S1.doc]

**Table S1:** Oligonucleotides used in this study.

| target | sequence | PCR no. in Fig. 3 |
| --- | --- | --- |
| 16SsRNA | agagtttgatcctggctc | control |
|  | cggctaccttgttacgac |  |
| *tcaB* | ggtgctgaagtcaacacc | 1 |
|  | aggaacttcctgactgcg |  |
| *tcaR1- tcaR2* | gtggtgaagtggaagtgc | 5 |
|  | agcgatccttcctgaacc |  |
| *tcaR2* | caggaaggatcgcttacc | 6 |
|  | ctatagatagtccagcacc |  |
| *tcaR2- tcaA* | actctcccctttcatggc | 7 |
|  | gctcagtttagtgatacc |  |
| *tcaA- tcaB1* | gggatcaccatagatgagg | 10 |
|  | ggtgttgacttcagcacc |  |
| *tcaB2- tcaC* | ctgttgcaaagcctgagc | 13 |
|  | gtggaacatcaagacttgc |  |
| *tcaC* | tcattatgagcgcatccc | 14 |
|  | aaggcaaattgtatctcgc |  |
| *tcaC*- ORF8 | aggatgaaaacgacaccg | 15 |
|  | cgatcaacactaagtcgg |  |
| ORF7- ORF8 | ggacttcttaatcctcgg | 16 |
|  | cgatcaacactaagtcgg |  |
| ORF8- ORF9 | cagttagttcgcaatggc | 17 |
|  | cgctctagctcttgaacc |  |
| ORF9- *tccC* | ggttcaagagctagagcg | 18 |
|  | cagcgttatagcggttgc |  |
| *tccC* | tcagactgatgccaaagg | 19 |
|  | ccatcaatagttcctgcc |  |
| *tccC*- ORF11 | actcgacctaacgagtcc | 20 |
|  | ggttcatcagcaatgacc |  |
| ORF11 | ggtcattgctgatgaacc | 21 |
|  | tccacatcaagcaaaccg |  |
| *tcaR1*-YE3798 | gctggaatacagtgtacg | 22 |
|  | ggtcaggtggatatcacc |  |
| ORF11-YE3798 | ggtcattgctgatgaacc | 34 |
|  | ggtcaggtggatatcacc |  |
| *tcbA* | cctcagttgcaacaagcc | 36 |
|  | gctacgctcaactgatgg |  |
| *tccC* | gctaacactcagcgatgg | 38 |
|  | cctaatgtgacccctacg |  |
| *tcaR1* | gcatgttgcaggaagtgc | 39 |
|  | cgtacactgtattccagc |  |
| *tcaA* | ccaatcaatgttctcacc | 46 |
|  | ggtgttgacttcagcacc |  |
| *tcYF1* | catccggcaacgtcac |  |
|  | ctggtacagaaactgcg |  |
| *tcYF2* | ctgttactgaccgtcc |  |
|  | gaacagcgtcgccgg |  |
| *tcYF3* | ctccgttactcgggac |  |
|  | cccggtgaaagcagg |  |
